# Supplementary material for: Association between oral microbiome and seven types of cancers in East Asian population: a two-sample Mendelian randomization analysis
Source: Front Mol Biosci. 2023 Nov 21;10:1327893. doi: 10.3389/fmolb.2023.1327893 (PMC10702768; doi:10.3389/fmolb.2023.1327893)
Supplement: Supplementary file 1 [file DataSheet1.ZIP › Supplementary Materials/MR plots of saliva/saliva═╝/colorectal cancer/pheno.598_to_colorectal cancer_leave_one_out.pdf]

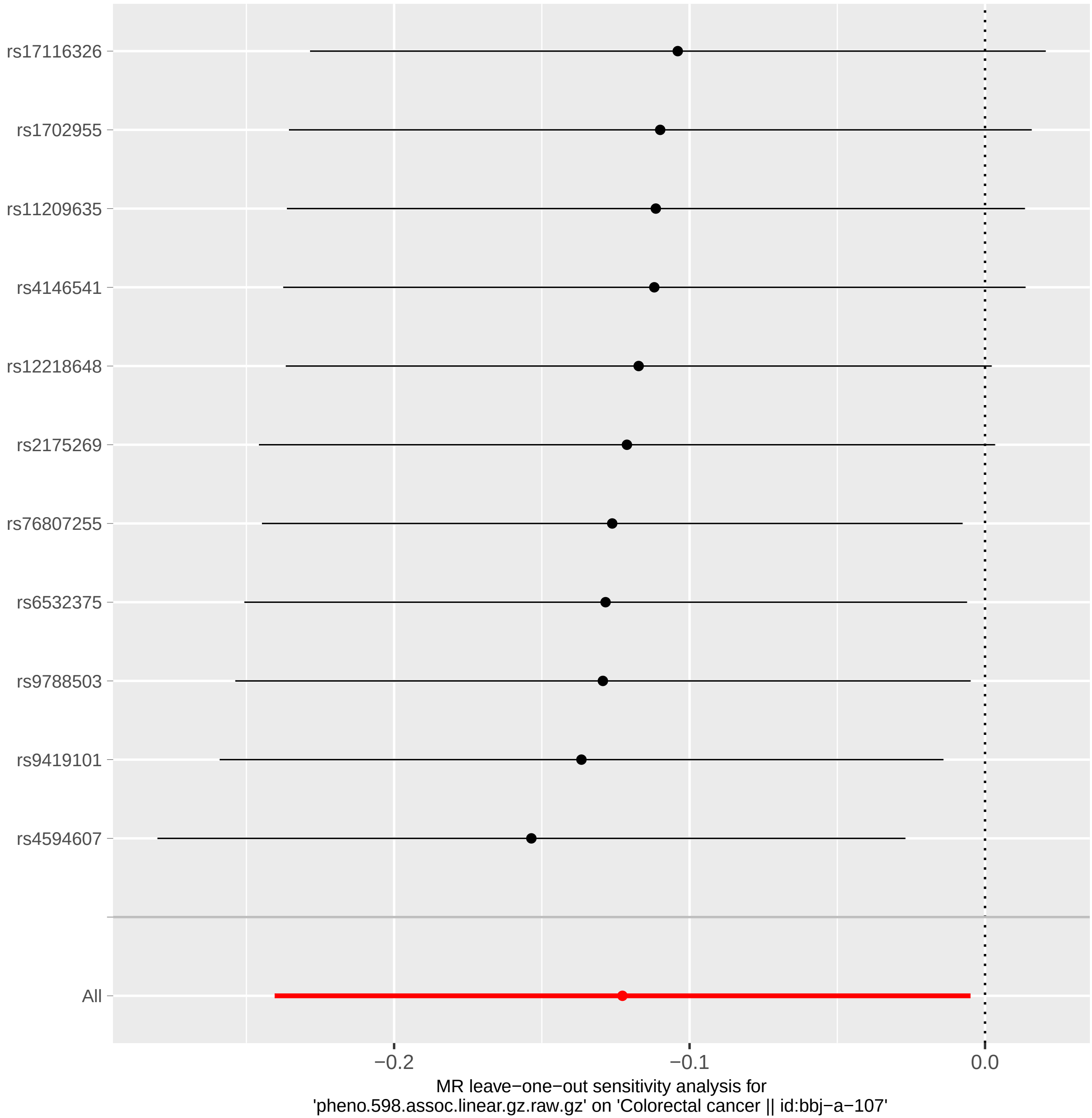

rs17116326
rs1702955
rs11209635
rs4146541
rs12218648
rs2175269
rs76807255
rs6532375
rs9788503
rs9419101
rs4594607
All
−0.2
−0.1
0.0
MR leave−one−out sensitivity analysis for
'pheno.598.assoc.linear.gz.raw.gz' on 'Colorectal cancer || id:bbj−a−107'
